# Open pollution routing problem of logistics distribution in medical union based on differential search algorithm

**DOI:** 10.1038/s41598-022-23387-3

**Published:** 2022-11-14

**Authors:** Xiaoxiao Quan, Yongsheng Pang, Jiansheng Chen, Xianghua Chu, Lina Shangguan

**Affiliations:** 1 Wuhan University, Wuhan, China; 2 Shenzhen Second People’s Hospital (The First Affiliated Hospital of Shenzhen University), Shenzhen, China; 3 College of Management, Shenzhen University, Shenzhen, China

**Keywords:** Environmental sciences, Environmental social sciences, Mathematics and computing

## Abstract

Medical care is a guarantee of people's daily life. Improving healthcare contributes to people's well-being. However, healthcare resources are characterized by uneven distribution. Financially well-off areas will have higher quality health care resources. Most of the medical resources are concentrated in public general hospitals, however, primary care institutions can hardly meet the growing needs of people. To solve this problem, Medical Union achieves efficient deployment of resources by integrating various medical institutions in the same area. In the process of logistics integration of the medical union, the scale of logistics distribution expands accordingly. Transportation vehicles have high operating costs and produce greenhouse gases in the process of distribution. The optimization of the driving path of logistics distribution vehicles can reduce the operating cost, fuel consumption and carbon emission. To solve this kind of decentralized and complex vehicle routing problem, this paper proposes a pollution routing problem model considering electrical vehicle usage, customer's soft time window expectation, open path and carbon cost. A modified Differential Search Algorithm with the comprehensive learning strategy and dynamic Cauchy variation strategy is advanced to solve the problem. Results show that the improved algorithm has good solving performance, and verifies the rationality of the proposed model, which will help to reduce carbon emissions and save the logistics and operating costs of medical devices.

## Introduction

Medical services are necessary to ensure a healthy life for people. However, the quantity and quality of medical resources available to people in different regions are not always the same, due to the different financial situations in different regions. In China, high-quality medical resources are concentrated in large public hospitals, but primary care institutions in communities cannot meet the demand for medical care. As a result, the medical union have been proposed to address the uneven layout of medical resources.

The sharing and deployment of supplies is a topic worth examining in a medical resource sharing program. Especially during the pandemic, large amounts of testing supplies are urgently needed in areas where cases have been identified. Since the medical union is trans-regional and scattered in distribution, some problems arise from gradual implementation of integrated management. To be specific, as logistics materials are more extensively distributed, massive fossil fuel has to be consumed to guarantee supply of those trans-regionally transported cargo. Carbon emission resulting from the process is affected by both cargo quantity and transportation routing. Therefore, optimized routing for logistics vehicles can not only bring down various distribution costs but also reduce fuel oil consumption and carbon emission.

Out of the consideration, how to plan a vehicle routing that can both save energy and reduce emission so as to cut down medical union operating costs has become a focus of much attention. It can be considered as a Vehicle Routing Problem (VRP). The VRP was first proposed by Dantzig and Ramser^1^ to find the best route for the vehicles and minimize general costs. However, in addition to the regular transportation costs, the level of tolerance of healthcare professionals for delayed delivery has to be considered in the medical setting. The soft time window constraint should be taken into account in the VRP.

In this study, the Mixed Vehicles Open Pollution Routing Problem with Time Window (TWMOPRP) is advanced. It takes into account such factors as EV, soft time window, open routing and carbon emission cost. TWMOPRP aims to minimize general cost while cutting down carbon emission, which is an NP-hard problem. What’s more, a modified Differential Search Algorithm (MDSA), a kind of swarm intelligent optimization algorithm, is proposed to solve the TWMOPRP. For the characteristics of the VRP problem, MDSA introduces two strategies. The results of simulation experiments showed that the TWMOPRP is in line with the reality and MDSA has a satisfactory solving performance.

## Related work

### Medical resource integration

Resource consolidation is a hot topic in healthcare today, especially at a time when local government budgets are limited. A tiered approach allows for the optimal allocation of resources at all levels of health and maximizes the use of medical resources^2^. The horizontal and vertical integration models are based on different forms of health care resource delivery. The horizontal integration model is the further integration of health care resources, while the vertical integration model is a combination of different levels and types of health care resources^3^.

There are a lot of studies about the integration of medical resource. According to Kodner and Spreeuwenberg^4^, health care resource integration is a partnership between the medical and health care sectors that needs to be achieved through the development of a mode for financing, service delivery, management, and medical capacity. While Vondeling^5^ defines health care resource integration as the planning, management, and delivery of a tightly integrated and coordinated set of systems to individual consumers through a range of organizations, collaborative professionals, and informal health care workers. Nowadays, a gradual shift occurred in the goal and focus of medical resource integration. The value chain is integrated by health as the goal, and the service process is structured in patient-centered.

The medical union breaks the original technical and administrative barriers through different levels and types of medical institutions, and finally integrates medical resources such as people, finance, materials and information into a common interest^6^. Therefore, the implementation of the construction of medical union is an important way to optimize the integration of medical resources. The study of medical union can provide some reference for the improvement of the theoretical system and the decision of governmental system innovation.

### Open pollution routing problem

As people are increasingly concerned about global climate change, researchers begin to consider greenhouse gases arising from oil-fueled vehicles in driving when examining traditional VRP and attempting to minimize cost while cutting down carbon emission. Based on VRP, Bektas and Laporte^7^ proposed Pollution Routing Problem (PRP), discussing the effect of the different influencing factors on carbon emission.

From then on, an increasing number of variables are incorporated into modeling so that the model could be as close to practical conditions as possible. Franceschetti et al.^8^ built a time-related PRP model and offered emission-reducing suggestions for solving speed limit issue in traffic jams. To find trade-offs between fuel consumption and driver times, Demir et al.^9^ proposed a bi-objective variant of the PRP and validated that significant reductions in carbon emissions can be achieved without major compromises in driving time. Furthermore, Koc et al.^10^ explored the impact of the heterogeneous fleet of vehicles on transportation costs and carbon emissions.

In 1981, the first study of the Open VRP was conducted by Schrage^11^. Open VRP means vehicles do not return to their original yard immediately after delivery. Various algorithms were proposed to solve the problem. Tarantilis et al.^12^ tried threshold accepting approach and found it is effective for fleet planning in real-life. Branch-and-cut algorithm also accepted to the problem and can provided a useful lower bound for large instances^13^. Fleszar et al.^14^ used a variable neighborhood search algorithm and obtained good performances in sixteen standard benchmark problem instances. What’s more, meta-heuristic algorithms, such as particle swarm optimization^15^, tabu search^16^ and ant colony optimization^17^, were also proven to be an effective and robust solution methods. What’s more, Hosseinabadi et al.^18^ proposed the OVRP_GELS and found it show a good performance than other existing algorithms.

Though having included fuel oil consumption or carbon emission based on VRP, previous studies on PRP are largely limited to gas vehicle (GV) in modeling. Few of them involve increasingly popular electric vehicle (EV). In recent years, the government has offered strong support for EVs. Lower after-subsidy retail price has attracted logistics enterprises to purchase EVs to carry out logistics business. Moreover, less environment pollution of EVs also satisfies “saving cost and reducing emission” demand put forward by supervisory authorities.

In the VRP study on EV, Liao et al.^19^ established an EV-VRP model involving carbon transaction cost to discuss difference in carbon emission between GV and EV. Schneider et al.^20^ considered the impact of the use of recharging stations on the routing problem. While Keskin and Çatay^21^ built the mathematical model for the electric VRP and found that the partial recharges can improve the solutions. Abdallah and Adel^22^ investigated the effect of variable speed on the VRP problem and argued that variable speed can solve the limitation of time window or distance. Therefore, each of the above-mentioned factors is also considered in the modeling of this paper.

### Differential search algorithm

Differential Search Algorithm (DSA)^23^ was proposed by Civicioglu to solve the conversion problem from geocentric right-angle coordinates to geodetic coordinates. It is a sort of swarm intelligence algorithm that simulates Brownian movement-like migrating activity in biotic communities. The previous study validated that DSA could be applied to global optimizing problems with better solving accuracy and convergence speed than many classical algorithms^24^.

Moreover, some researchers have applied DSA to solve some practical problems. Gui et al.^25^ proposed a hybrid differential search algorithm (HDSA) to optimize the medical image alignment problem and compared the effectiveness with the standard algorithm. Chu et al.^26^ advanced a new model of cross-training with learning and forgetting effects, and presented an adaptive DSA to solve the problem of worker assignment across multiple units. Ma et al.^27^ built a dynamic resource allocation model based on cost optimization and SLA constraints and designed an improved DSA to accurately predict the load. Al-Fakih et al.^28^ presented an improved binary DSA for QSAR classification of multiple series of antibacterial compounds with Candida albicans inhibitors. DSA can be found to be used effectively in various fields, which also confirms its applicability and validity.

To improve the DSA for various problems, researchers proposed some new strategies to improve search capabilities and combined different techniques to avoid premature convergence. Liu^29^ proposed four search schemes to speed up the convergence by searching the new space of the global optimization problem. To improve the convergence speed and solution quality, Guha^30^ introduced a learning method based on proposed opposition. Chen et al.^31^ used a Latin hypercube sampling method for initialization and combined DSA with simplex methods for search. Furthermore, the low-frequency oscillation problem was solved by combining the search direction based on random permutation of the original population with DSA^32^. By introducing different search strategies helps to improve the performance of algorithm on different problems^33^, so two strategies for DSA are considered and their effectiveness is experimentally confirmed in this paper.

## Problems description and model building

### Problems description

Defined on a directed graph *G* = {*N*, *A*}, TWMOPRP is composed of a set of nodes *N* = {0, 1, 2, …, *n*} and a set of inter-nodal arcs *A*. Among the nodes, node 0 indicates distribution center. Suppose a logistics distribution company owns several EVs and GVs which both have a capacity of *Q*. Due to shorter range per charge, EV cannot completely substitute GV. Therefore, logistics companies prefer the combination of GV and EV in the short run. The client set *N*_*0*_ = *N*\{0} is composed of *n* clients, each of which *i* ∈ *N*_*0*_ has demand *q*_*i*_ and soft distribution time window [*a*_*i*_, *b*_*i*_]. Distribution outside time window would impair both parties’ actual benefits and thus incur matching penalty cost. GV is a source of carbon emission in driving. In consideration of the possible release of carbon emission policy by central government, carbon emission cost may be converted to carbon cost. By comparison, EV has no such expenditure. In case of open routing, after completing a distribution task, vehicles won’t return to the original distribution center but head to the nearest distribution center to start a new task or take a break. Based on traditional PRP, TWMOPRP takes into account more factors related to logistics distribution process so that the model could be better applied to actual application.

### Model hypotheses and symbol description

TWMOPRP is preconditioned on the following hypotheses:

1. Since the model considers only a small period of time that starts with departure from distribution center and ends with completion of distribution, only one distribution center is designed.
2. Such information as client demand and distribution time window remains known and constant during distribution.
3. Each client can only be accessed once and optimal distribution time window is known. Distribution vehicle should strive to complete the distribution within the time window specified by client, and the client also accepts the distribution outside the time window.
4. Logistics company owns a certain number of GVs and EVs that could meet distribution demand.
5. Vehicle runs at a constant average speed, there is no slope to be considered along the distribution route and there is no unexpected situation that could affect distribution efficiency such as traffic jam.
6. Cargo loaded on vehicle along the trip could not exceed maximum capacity, but the vehicle could run on full load.
7. EV departs from distribution center with full charge and electricity consumed during cargo handling at client site is negligible. No charging or power switching is conducted during distribution trip.

Table 1 details the variables and parameters involved in the TWMOPRP model proposed in this paper.

**Table 1:** Variables and parameters used in the model.

Symbol	Description
*N*	Set of all nodes
*N*_*0*_	Set of client nodes
*K*	Set of vehicles owned by company
*d*_*i,j*_	Distance from client i to j
*v*	Average running speed of vehicle
*s*_*i*_	Service hours at client i
*t*_*i*_	Time when vehicle arrives at client i
*S*_*j*_	All time required by vehicle to arrive at last client j
*E*_*max*_	Max battery capacity of EV
*σ*	Power consumed by EV for each km
*e*_*j*_	Residual power of EV at client j
*ET*_*i*_	Earliest time permissible for vehicle to arrive at client j
*LT*_*i*_	Latest time permissible for vehicle to arrive at client j
*Q*	Max. vehicle load
*q*_*i*_	Demand of client i
*f*_*ij*_	Freight of vehicle from client i to j
*C*_*1*_	GV’s transportation cost for unit distance
*C*_*2*_	EV’s transportation cost for unit distance
*T*	Tax price for unit carbon emission
*P*	Driver’s hourly salary
*pe*	Unit penalty cost coefficient for earlier arrival than expected
*pl*	Unit penalty cost coefficient for later arrival than expected

### PRP model with GV only

1 $$\begin{gathered} \min Z = \sum\limits_{{i \in N,j \in N_{0} ,i \ne j}} {\sum\limits_{k \in K} {C_{1} d_{ij} x_{ijk}^{C} } } + \sum\limits_{{i \in N,j \in N_{0} ,i \ne j}} {\sum\limits_{k \in K} {C_{ij} x_{ijk}^{C} } } + \sum\limits_{{j \in N_{0} }} {\sum\limits_{k \in K} {PS_{jk} } } \hfill \\ + \sum\limits_{i \in N} {\max \left\{ {pe(ET_{i} - t_{i} );0;pl(t_{i} - LT_{i} )} \right\}} \hfill \\ \end{gathered}$$

2 $${\text{s}}.{\text{t}}.\sum\limits_{{j \in N_{0} }} {\sum\limits_{k \in K} {x_{ijk}^{C} = 1} } ,\quad \forall \,\,i \in N;$$

3 $$\sum\limits_{i \in N} {\sum\limits_{k \in K} {x_{ijk}^{C} = 1} } ,\quad \forall j \in N_{0} ;$$

4 $$\sum\limits_{j \in N} {f_{ji} } - \sum\limits_{j \in N} {f_{ij} } = q_{i} ,\quad \forall j \in N_{0} ;$$

5 $$q_{j} x_{ijk}^{C} \le f_{ij} \le (Q - q_{i} )x_{ijk}^{C} ,\,\,\,\forall i,j \in N_{0} ,i \ne j,k \in K;$$

6 $$f_{ij} \ge 0,\quad \forall i,j \in N_{0} ,i \ne j;$$

7 $$\sum\limits_{i \in N} {\sum\limits_{{j \in N_{0} }} {q_{j} x_{ijk}^{C} \le Q} } ,\quad \forall k \in K;$$

8 $$t_{j} = t_{i} + s_{i} + \frac{{d_{ij} }}{v},\,\,\,\forall i \in N,j \in N_{0} ;$$

9 $$x_{ijk}^{C} \in \left\{ {0,1} \right\},\quad \forall i \in N,j \in N_{0} ,i \ne j,k \in K$$

where Eq. (1) is an objective function that is made up of four items, namely vehicle transportation cost, carbon cost, driver salary and time window penalty cost. Equations (2)–(9) are constraints. Equations (2) and (3) mean each client is and can only be accessed once. Equation (4) means vehicle keeps same freight volume when accessing client. Equations (5)–(7) are to make sure max vehicle load is always above client demand during transportation. Equation (8) is to figure out the time span from accessing no. *i* client to reaching no. *j* client of the vehicle. Equation (9) is a decision variable. When $x_{ijk}^{C} = 1$, the no. *k* GV will depart from client *i* to *j*. If the value is 0, it does not move.

In this paper, integrated fuel emission model proposed by Barth et al.^34^ is employed to determine fuel oil consumption rate FR (unit: g/s) of GV. The model is demonstrated using the following formula:

10 $$P_{t} = (Ma + Mg\sin \theta + 0.5C_{d} A\rho v^{2} + MgC_{r} \cos \theta )v/1000$$

11 $$FR = \xi (kNV + (P_{t} /\varepsilon + P_{a} )/\eta )/\mu$$

where *P*_*t*_ indicates engine traction power, *P*_*a*_ indicates additional engine power demand which is usually supposed to be 0.

Suppose GV runs along arc (*i, j*) with a distance of *d*_*i,j*_, vehicle runs at an average speed of *v*_*i,j*_, carbon price is *T* Yuan/kg, the carbon emission cost on this trip will be:

12 $$C_{ij} = cFR\frac{{d_{ij} }}{{v_{ij} }}T$$

### TWMOPRP model with mixed vehicles

TWMOPRP model takes into account the scenario of introducing EV into cargo transportation. Different from GV, EV is set with lowest electric quantity threshold in the model. When vehicle’s actual electric quantity is lower than this threshold, it should go to nearest distribution center for charging or replacing. Thus, in assigning distribution tasks, the model takes into consideration the power consumption of EV so that EV has residual power higher than safety threshold after completing single task. TWMOPRP model is shown below:

13 $$\begin{gathered} \min Z = \sum\limits_{{i \in N,j \in N_{0} ,i \ne j}} {\sum\limits_{k \in K} {d_{ij} (C_{1} x_{ijk}^{C} } } + C_{2} x_{ijk}^{E} ) + \sum\limits_{{i \in N,j \in N_{0} ,i \ne j}} {\sum\limits_{k \in K} {C_{ij} x_{ijk}^{C} + CE_{ij} x_{ijk}^{E} } } \hfill \\ + \sum\limits_{{j \in N_{0} }} {\sum\limits_{k \in K} {PS_{jk} } } + \sum\limits_{i \in N} {\max \left\{ {pe(ET_{i} - t_{i} );0;pl(t_{i} - LT_{i} )} \right\}} \hfill \\ \end{gathered}$$

14 $${\text{s}}.{\text{t}}.\sum\limits_{{j \in N_{0} }} {\sum\limits_{k \in K} {(x_{ijk}^{C} + x_{ijk}^{E} ) = 1} } ,\quad \forall i \in N;$$

15 $$\sum\limits_{i \in N} {\sum\limits_{k \in K} {(x_{ijk}^{C} + x_{ijk}^{E} ) = 1} } ,\quad \forall j \in N_{0} ;$$

16 $$\sum\limits_{j \in N} {f_{ji} } - \sum\limits_{j \in N} {f_{ij} } = q_{i} ,\quad \forall j \in N_{0} ;$$

17 $$q_{j} (x_{ijk}^{C} + x_{ijk}^{E} ) \le f_{ij} \le (Q - q_{i} )(x_{ijk}^{C} + x_{ijk}^{E} ),\quad \forall i,j \in N_{0} ,i \ne j,k \in K;$$

18 $$f_{ij} \ge 0,\quad \forall i,j \in N_{0} ,i \ne j;$$

19 $$\sum\limits_{i \in N} {\sum\limits_{{j \in N_{0} }} {q_{j} (x_{ijk}^{C} + x_{ijk}^{E} ) \le Q} } ,\quad \forall k \in K;$$

20 $$e_{0k} x_{0jk}^{E} = E_{\max } ,\quad \forall j \in N_{0} ,k \in K;$$

21 $$e_{j} \le (e_{i} - \sigma d_{ij} )x_{ijk}^{E} + E_{\max } (1 - x_{ijk}^{E} ),\quad \forall i \in N,j \in N_{0} ,k \in K;$$

22 $$e_{ik}^{a} x_{ik}^{E} = e_{ik}^{g} x_{ik}^{E} ,\quad \forall i \in N,k \in K;$$

23 $$e_{j} \ge 0.25E_{\max } ,\quad \forall j \in N_{0} ;$$

24 $$t_{j} = t_{i} + s_{i} + \frac{{d_{ij} }}{v},\quad \forall i \in N,j \in N_{0} ;$$

25 $$x_{ijk}^{C} ,x_{ijk}^{E} \in \left\{ {0,1} \right\},\quad \forall i \in N,j \in N_{0} ,i \ne j,k \in K$$

where Eq. (13) is an objective function, Eqs. (14)–(25) are constraints. Equations (14) and (15) represent each client is and can only be accessed once and is to be served by one vehicle model. According to Eq. (20), EV departs from distribution center with full power. In Eq. (21), when shifting from client *i* to *j*, EV consumes power for driving purposes only. Equation (22) means EV doesn’t consume power when serving client *i*. Equation (23) constrains the lowest power consumed for driving vehicle. Equation (25) is a decision variable. When $x_{ijk}^{E} = 1$, the no. *k* EV will depart from client *i* to *j*. If the value is 0, it does not move.

Because EV uses electricity exclusively, they do not produce direct carbon emissions while driving. Considering that it is still dominated by thermal power generation in China, the carbon emissions generated during power generation should be taken into account in the carbon emissions of EV. Suppose it runs along arc (*i, j*) with a distance of *d*_*i,j*_ and the electricity emission factor is *e*, the carbon cost on this trip will be:

26 $$CE_{ij} = e\sigma d_{ij}$$

## Solving algorithms

### Differential search algorithm

DSA simulates the spontaneous migratory process of biotic communities in natural world to obtain richer resources. Adopting two steps “look for optimal location and midway temporary stay”, it aims to find the optimal habitat. In DSA, *X*_*i*_ (*i* = 1, 2, …, *N*) indicates no. *i* biological organism, *x*_*i,j*_(*j* = 1,2,…,*D*) indicates location of no. *i* biological organism on no. *j* dimension, and several bio-organisms could form a Superorganism. According to the algorithm, *N* indicates number of bio-organisms within the space, while *D* indicates dimension of actual problem. DSA works in the following process:

In the first place, several bio-organisms are randomly initialized in terms of their locations on all dimensions:

27 $$x_{ij} = lb_{j} + {\text{rand}}(ub_{j} - lb_{j} )$$

where *rand()* is a random number within [0,1] range. Those bio-organisms could form a superorganism. After being initialized, *Superorganism* = [*X*_*i*_] = [*x*_*i,j*_] would be generated with a new habitat based on:

28 $$S_{i} = X_{i} + Scale(donor - X_{i} )$$

where *donor* indicates the new direction after random change of original individual sequence with following definition *donor* = [*X*_*random_shuffling(i)*_]; Scale indicates movement step size generated with one gamma random inventor as defined below:

29 $$Scale = randg[2rand_{1} ](rand_{2} - rand_{3} )$$

Finally, the bio-organism would evaluate whether the new location is more fertile than the original one so that it could choose forwarding to a new habitat or keeping still according to a Greedy Policy in Eq. (30). With changing locations of bio-organisms, superorganism gradually approach global optimal location until the algorithm’s terminal condition is met.

30 $$X_{i,T + 1} = \left\{ {\begin{array}{*{20}c} {X_{i,T} ,f(X_{i,T} ) < f(S_{i,T} )} \\ {S_{i,T} ,f(X_{i,T} ) \ge f(S_{i,T} )} \\ \end{array} } \right.$$

### Algorithm improvement strategy

Employing Brownian movement-like search strategy, traditional DSA has higher randomness and thus wider algorithm search scope. However, it also makes it hard for the algorithm to achieve convergence in later stage. Out of this consideration, in this paper, two strategies are introduced to improve DSA’s solving capacity.

#### Comprehensive learning strategy

Liang et al.^35^ proposed Comprehensive Learning Strategy that could modify Particle Swarm Optimization Algorithm by updating speed based on historical optimal data of other particles. The strategy could maintain colonial diversity and avoid being caught in local optimal. In this paper, Comprehensive Learning Strategy is introduced into DSA to learn after individual historical optimal or other’s historical optimal with probability choice. MDSA generates new habitat using:

31 $$S_{i} = X_{i} + Scale(pbest_{{f_{i} }} - X_{i} )$$

where *f*_*i*_ = [*f*_*i*_(1), *f*_*i*_(2), …, *f*_*i*_(*D*)] indicates the object available to be learnt by no. *i* bio-organism, *pbest*_*fi*_ indicates a set of learning objects with established order. On each dimension, it could be itself or other bio-organisms, which are decided by probability value *Pr*. Pseudocode of Comprehensive Learning Strategy is demonstrated in Algorithm 1 in which *fit*() indicates fitness of habitat, whereas *randi*() indicates a random integer generated from uniform discrete distribution.
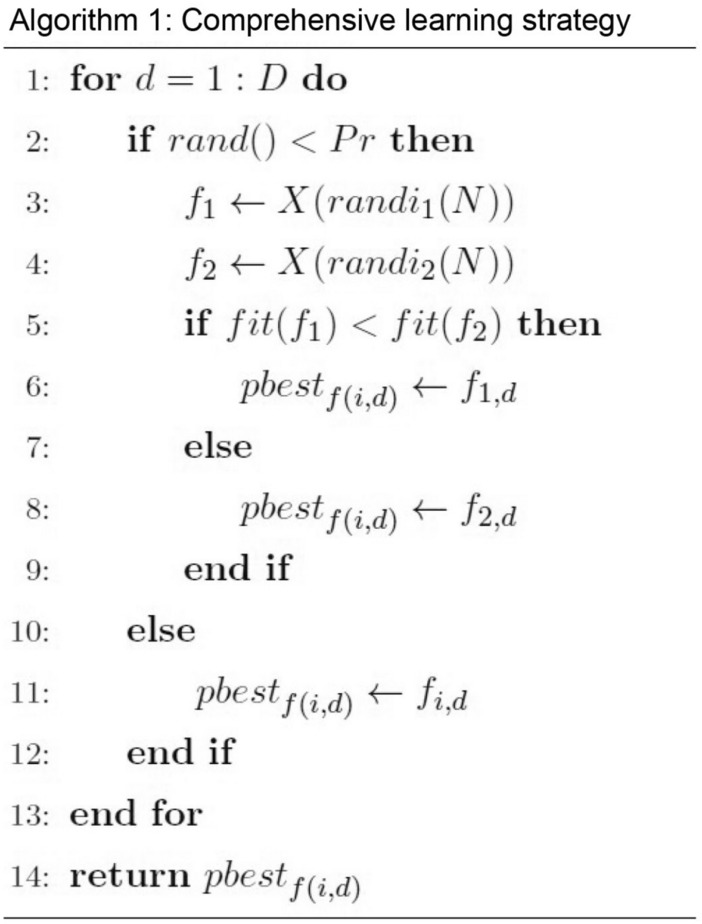


#### Dynamic Cauchy mutation-based evolutionary strategy

Yao and Liu^36^ came up with a sort of evolutionary strategy based on Cauchy Mutation to strengthen colonial diversity of algorithm and get rid of local optimal, but it has common search performance when implemented in local small neighborhood. To cope with this problem, in this paper a dynamic Cauchy mutation strategy is suggested to speed up the convergence of algorithm in the later stage. The strategy is defined as below:

32 $$X_{i,d}^{t^{\prime}} = X_{g,d}^{t} + \alpha {\text{cauchy}}(\delta )$$

where $X_{g,d}^{t}$ is the optimal fitness value, *cauchy*(*δ*) is a random number generated with Cauchy distribution function with ratio parameter *δ* being 1, and α indicates step size in search by algorithm as defined below:

33 $$\alpha = 1 - rand^{\frac{t}{T}}$$

where *t* indicates the present number of iterations and *T* indicates the general number of iterations.

### Modified DSA

**Figure 1:**
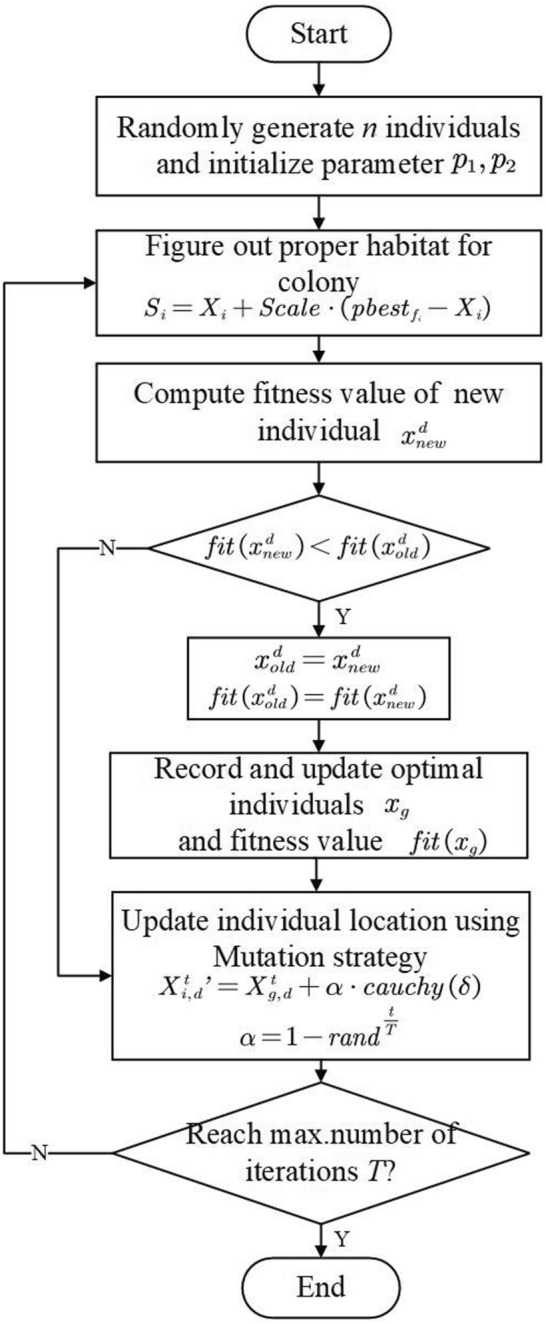
Schematic diagram of MDSA algorithm.

The aforesaid two improvement strategies are applied to traditional DSA algorithm in order to prevent hard convergence and local optimal solution of algorithm. MDSA proposed in this paper has the following solving process (Fig. 1).

## Experiments and analysis

### Experiments setting

To verify the effectiveness of TWMOPRP model and MDSA algorithm proposed in this paper, we have generated three different sizes of simulation data with Solomon example, as well as their demand and time windows in random. They have 10, 30, and 50 simulated clients. Distribution center is supposed to be located at the center of a 80 × 80 km rectangle and all clients are to be within the distribution range. Assume that all the vehicles start their delivery tasks from 8:00 a.m. and the moment is recorded as zero minute. The detail of clients and the parameters used can be found in the supplementary file S1.

### Results and interpretation

#### Comparison of algorithms

To validate the performance of MDSA, three other algorithms, ABC, AMBSO and CLPSO, are selected to conduct a comparative analysis. ABC^37^ is an optimization algorithm with a swarm as the base population, which has a fast convergence rate and a strong ability to get rid of local optimal solutions. AMBSO^38^ is the improved version of the brainstorm optimization algorithm, another swarm intelligence algorithm inspired by the human problem-solving process, with two complementary strategies. While CPLSO^35^ is the modified edition of Particle Swarm Optimization algorithm with comprehensive learning strategy.

Those algorithms are set to work on a colony of 100 and for 1,000 iterations at most in solving TWMOPRP. The other parameters use the recommended values from the original literatures. The experiments are implemented on Matlab 2016b. Each experiment is repeated 30 times and the average value was used as the final result. The results of the comparison are shown in Table 2.

**Table 2:** The performances of different algorithms in solving TWMOPRP.

Problem	Algorithm	Total cost (Yuan)	Carbon emission (kg)	CPU processing (s)
10 clients	MDSA	886.84	123.10	65.34
	ABC	994.63	187.27	87.81
	AMBSO	963.68	156.84	79.01
	CLPSO	1026.38	173.36	85.91
30 clients	MDSA	1637.59	225.18	114.02
	ABC	1817.49	319.08	156.26
	AMBSO	1735.82	275.11	138.61
	CLPSO	1857.58	330.04	152.83
50 clients	MDSA	2321.47	327.77	161.03
	ABC	2899.01	458.62	184.39
	AMBSO	2674.93	432.67	227.92
	CLPSO	2744.24	485.06	209.25

**Figure 2:**
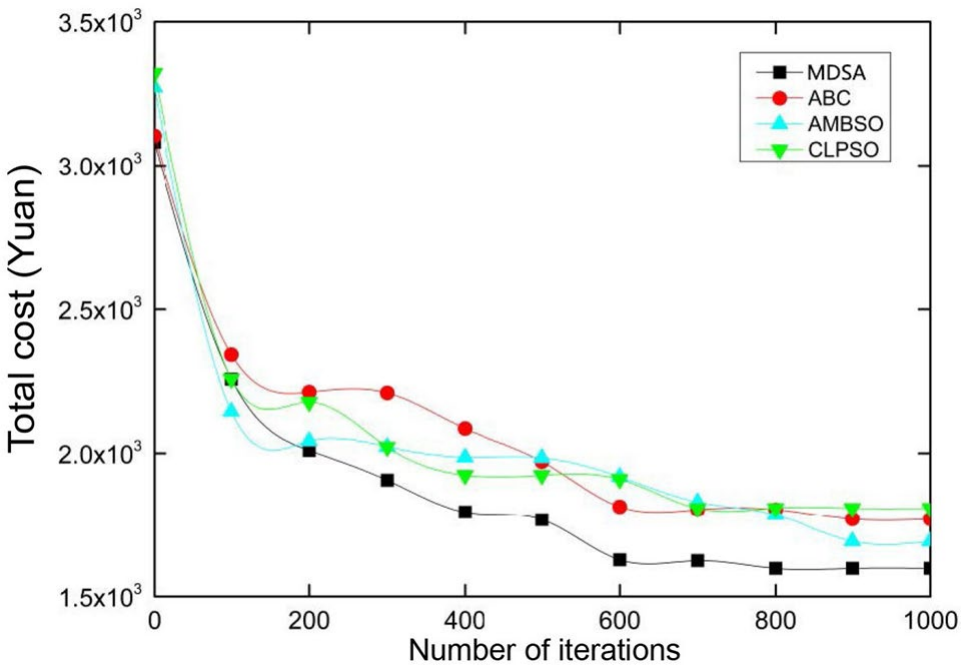
Comparison of algorithms in convergence.

It is not difficult to find that MDSA stands out in all indicators when compared with the other three algorithms. Its total cost in problem solving is about 11% lower, its carbon emission is about 30% lower and its solving process is about 26% faster than others. Figure 2 illustrates convergence of all algorithms in solving TWMOPRP, according to which MDSA is the quickest in search and best in solution quality. Findings suggest MDSA is suitable for solving complicated VRP.

#### Analysis of factors affecting carbon emission

To explore the effect of EV, vehicle running speed, carbon price and driver salary on carbon emission arising from logistics distribution, this paper employs MDSA to solve GV-only PRP model and mixed TWMOPRP model, respectively. Each solving process is repeated 30 times and average value of each indicator is taken as the final result in analysis. Experimental findings are listed below.

1. Effect of vehicle model in use.

Since EV faces several unfavorable constraints such as limited battery capacity and charging in use, whether GV should be entirely replaced with EV still needs further discussion. The results of the effect of various vehicles are shown in Table 3.

**Table 3:** The effect of various vehicles.

Model used	Total cost (Yuan)	Time window cost (Yuan)	Carbon emission (kg)
Mixed	1628.52	199.36	264.65
GV	1632.53	206.17	281.44

When EV is introduced, both total cost and time window cost decline slightly, and carbon emission decreases more significantly. The carbon emission directly resulting from EV is lower, so the carbon cost from single distribution is also lower. EV could help bring down total carbon emission in logistics and meet the demand of saving energy and reducing emission when being used in freight distribution. In addition, it could also indirectly cut down operating cost of the medical union.

(2) Effect of vehicle speed.

A study by Bektas and Laporte^7^ indicates speed of distributing vehicle could affect its carbon emission when there is time window limit. Furthermore, vehicle speed could also affect distribution cost and customer satisfaction. Therefore, it is critical to choose a proper speed during distribution. The results of the effect of various speeds are shown in Table 4.

**Table 4:** The effect of various speeds.

Speed (km/h)	Time window cost (Yuan)	Driver cost (Yuan)	Carbon emission cost (Yuan)	Carbon emission (kg)
40	232.18	542.54	153.64	307.28
50	202.76	492.87	138.29	276.58
60	199.36	453.22	134.77	269.54
70	249.47	414.86	146.56	293.12

**Figure 3:**
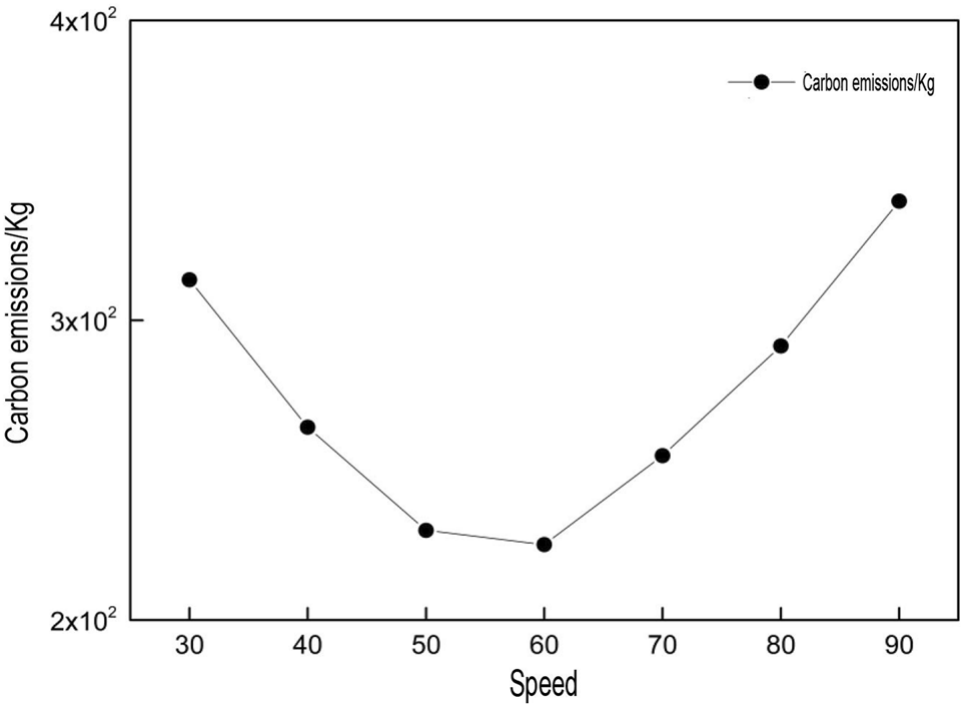
Effect of various speeds on carbon emission.

The table above makes it clear that, when vehicle runs at 40 km/h, highest level of carbon emission as affected by driver salary and distribution is witnessed. As speed keeps rising, all indicators are found to rise first and then fall. They appear to be better when vehicle speed is 60 km/h. Figure 3 shows the carbon emission arising from vehicles running at various speeds. As shown in the figure, over high or low speed brings in more carbon emissions. For this reason, when vehicle runs at a proper speed during the trip, it could meet the requirements of energy saving and emission reduction. In further study, the effect of limited speed on distributing routing plan due to traffic jams and so on may be examined when carbon emission is taken into account.

(3) Effect of carbon price.

Carbon price is a decisive factor in affecting carbon emission cost as thus total distribution cost of medical union. Reasonable setting of carbon price is conducive in guiding logistics companies to offer distribution services with low carbon emission means. Nevertheless, the external effect of carbon price varies with its specific levels. In this paper, an analysis of the effect of carbon price on all indicators is performed. Corresponding findings are listed as below. The results of the effect of various carbon prices are shown in Table 5.

**Table 5:** The effect of various carbon prices.

Carbon price (Yuan/kg)	Time window cost (Yuan)	Driver cost (Yuan)	Carbon emission cost (Yuan)	Carbon emission (kg)
0.1	195.78	445.25	56.75	567.50
0.5	199.36	453.22	137.98	275.96
1	215.27	459.48	232.83	232.83
6	219.96	482.27	997.29	166.22
10	226.72	511.70	1377.49	137.75

When carbon price is raised, carbon emission decreases significantly, but other costs such as time window and driver rise slowly. It is infeasible to raise carbon price alone, because it may cause an increase in several other costs of enterprises as well as logistics distribution costs of the medical union. In the meanwhile, levying carbon emission cost is to guide companies to use EV in executing distribution tasks. However, EV use in freight transportation is still met with some inconveniences. Excessive EV using may undermine satisfaction with the medical union as well as the distribution company’s production efficiency. As a result, the government is suggested setting a reasonable carbon price that benefits all involved parties based on the status quo of local logistics industry and EV industry so that logistics industry could be encouraged to save energy and reduce emissions.

(4) Effect of driver salary.

Driver is a major participant in logistics distribution tasks. Change in labor cost may cause logistics distribution companies to change their operating decisions. In this paper, we tentatively discuss the correlation between driver’s hourly salaries and carbon emissions. The results of the effect of various salaries are shown in Table 6.

**Table 6:** The effect of various salaries.

Driver’s hourly salary (Yuan)	Time window cost (Yuan)	Driver cost (Yuan)	Carbon emission cost (Yuan)	Carbon emission (kg)
20	199.36	453.22	137.84	275.68
25	206.65	549.06	146.25	292.50
30	215.19	654.3	153.68	307.12

We can easily discover that raising driver’s hourly salary could increase total distribution cost and carbon emission as well. The reason is that over-high labor cost makes logistics distribution companies more willing to use GV featuring longer mileage and more flexible energy supply in distribution. In the logistics distribution process, the medical union should take into account as many key indirect factors as possible, such as labor cost and tax, so that indirect costs arising from logistics distribution integration could be raised and medical union’s benefits could be maximized.

## Conclusions

In this paper, Open VRP for medical supplies distribution in the context of medical union is discussed. Considering that the procurement of materials in medical institutions are preceded by medical services, a certain degree of delay in distribution is acceptable, but it causes problems for the material managers. Therefore, a soft time window constraint is taken into the TWMOPRP. In addition, with the growing call for "Net Zero", the logistics industry, as an industry that generates large amounts of carbon emissions, should take the lead in reducing greenhouse gas emissions.

On one hand, TWMOPRP introduces a penalty term for the cost of carbon emissions in order to reduce them when solving for a suitable route. On the other hand, considering the use of electric vehicles can also reduce direct carbon emissions and improve service satisfaction. After combining the above factors, the experimental results show that TWMOPRP has a strong rationality and is more in line with the realistic situation.

To solve TWMOPRP, this paper introduces a comprehensive learning strategy and a dynamic Cauchy Mutation-based evolutionary strategy based on DSA, and proposes MDSA. These two strategies enhance the search capability of DSA and accelerate the convergence speed. Experimental results show that MDSA has better solution performance for TWMOPRP than other similar algorithms, and the convergence speed is reduced by about 26%.

On the results of the MDSA solution, this study further analyzed the four influencing factors, and the following conclusions were obtained. The using of EV, maintaining 60 km/h speeds and lower driver hourly salaries help reduce cost and carbon emissions. Setting a reasonable price for carbon emissions will help to reduce carbon emissions without putting a large burden on logistics companies.

In further studies, more factors of EVs should be considered into TWMOPRP, such as the time and speed of charging. In addition, Open VRP for a single yard is a simplified model, which is closer to the actual situation if multiple yards are considered. To get reasonable solutions, more strategies can be taken into consideration to improve the performance of DSA.

## Supplementary Information

Supplementary Information.

## Data Availability

The datasets used during the current study are available in the supplementary file and from the corresponding author on reasonable request.
