# Supplementary material for: Open pollution routing problem of logistics distribution in medical union based on differential search algorithm
Source: Sci Rep. 2022 Nov 14;12:19472. doi: 10.1038/s41598-022-23387-3 (PMC9663830; doi:10.1038/s41598-022-23387-3)
Supplement: Supplementary file 1 — Supplementary Information. [file 41598_2022_23387_MOESM1_ESM.docx]

**Table 1. The information and demand of 10 clients.**

| **No.** | **X coordinate** | **Y coordinate** | **Demand/t** | **Time window/min** | |
| --- | --- | --- | --- | --- | --- |
| 0 | 40 | 40 | - | - | |
| 1 | 10.18 | 75.27 | 1.0 | 80 | 200 |
| 2 | 17.23 | 70.75 | 1.2 | 70 | 280 |
| 3 | 23.26 | 24.33 | 1.5 | 50 | 180 |
| 4 | 37.34 | 13.53 | 1.8 | 10 | 260 |
| 5 | 38.68 | 46.20 | 0.2 | 120 | 130 |
| 6 | 46.74 | 32.59 | 0.1 | 10 | 150 |
| 7 | 49.11 | 10.21 | 0.9 | 20 | 170 |
| 8 | 55.64 | 27.48 | 0.5 | 130 | 280 |
| 9 | 69.45 | 52.70 | 1.6 | 60 | 120 |
| 10 | 77.55 | 51.31 | 0.4 | 20 | 170 |

**Table 2. The information and demand of 30 clients.**

| **No.** | **X coordinate** | **Y coordinate** | **Demand/t** | **Time window/min** | |
| --- | --- | --- | --- | --- | --- |
| 0 | 40 | 40 | - | - | |
| 1 | 11.21 | 46.78 | 0.9 | 90 | 220 |
| 2 | 12.14 | 56.28 | 1 | 150 | 300 |
| 3 | 12.99 | 31.53 | 0.8 | 30 | 160 |
| 4 | 14.31 | 50.19 | 0.2 | 90 | 210 |
| 5 | 14.45 | 60.92 | 1.2 | 40 | 120 |
| 6 | 21.34 | 7.46 | 0.6 | 50 | 130 |
| 7 | 22.09 | 13.86 | 0.8 | 40 | 130 |
| 8 | 24.13 | 72.34 | 0.4 | 30 | 130 |
| 9 | 24.75 | 15.09 | 1.3 | 0 | 90 |
| 10 | 24.94 | 47.98 | 1.3 | 60 | 160 |
| 11 | 26.01 | 66.66 | 1.2 | 110 | 200 |
| 12 | 26.04 | 52.25 | 0.8 | 110 | 210 |
| 13 | 28.48 | 45.42 | 0.4 | 70 | 160 |
| 14 | 32.03 | 12.99 | 1.4 | 150 | 230 |
| 15 | 33.01 | 48.62 | 0.8 | 90 | 180 |
| 16 | 34.27 | 27.00 | 1 | 90 | 200 |
| 17 | 35.75 | 58.08 | 0.8 | 140 | 240 |
| 18 | 36.5 | 64.39 | 1.7 | 130 | 230 |
| 19 | 48.13 | 31.97 | 1.1 | 180 | 270 |
| 20 | 50.14 | 62.70 | 1.4 | 10 | 120 |
| 21 | 50.92 | 35.08 | 0.9 | 110 | 200 |
| 22 | 54.67 | 79.53 | 1.9 | 120 | 210 |
| 23 | 57.16 | 44.22 | 0.6 | 30 | 120 |
| 24 | 65.20 | 61.11 | 1.8 | 90 | 180 |
| 25 | 74.24 | 53.15 | 0.5 | 120 | 180 |
| 26 | 76.21 | 34.53 | 1.6 | 30 | 120 |
| 27 | 76.43 | 41.91 | 0.8 | 90 | 270 |
| 28 | 78.79 | 20.33 | 0.3 | 90 | 180 |
| 29 | 79.8 | 27.90 | 0.9 | 130 | 280 |
| 30 | 79.9 | 18.62 | 1.2 | 70 | 170 |

**Table 3. The information and demand of 50 clients.**

| **No.** | **X coordinate** | **Y coordinate** | **Demand/t** | **Time window/min** | |
| --- | --- | --- | --- | --- | --- |
| 0 | 40 | 40 | - | - | |
| 1 | 11.01 | 75.19 | 0.3 | 110 | 210 |
| 2 | 11.03 | 29.94 | 1.2 | 90 | 140 |
| 3 | 11.58 | 23.25 | 1.7 | 130 | 210 |
| 4 | 12.08 | 25.29 | 1.2 | 70 | 120 |
| 5 | 13.92 | 59.32 | 0.5 | 100 | 150 |
| 6 | 15.56 | 41.49 | 0.4 | 110 | 160 |
| 7 | 15.65 | 23.56 | 1.0 | 70 | 180 |
| 8 | 17.38 | 24.97 | 1.9 | 120 | 140 |
| 9 | 18.14 | 33.78 | 0.6 | 80 | 90 |
| 10 | 22.55 | 15.42 | 0.5 | 30 | 130 |
| 11 | 23.07 | 58.72 | 1.4 | 50 | 170 |
| 12 | 26.18 | 29.84 | 0.5 | 20 | 190 |
| 13 | 27.94 | 11.66 | 1.3 | 70 | 180 |
| 14 | 28.93 | 56.67 | 0.8 | 90 | 120 |
| 15 | 32.18 | 74.78 | 0.2 | 140 | 90 |
| 16 | 35.77 | 49.22 | 0.2 | 130 | 140 |
| 17 | 35.97 | 76.43 | 0.1 | 60 | 70 |
| 18 | 39.80 | 70.43 | 1.7 | 140 | 230 |
| 19 | 42.57 | 45.32 | 1.5 | 110 | 200 |
| 20 | 43.01 | 79.26 | 0.1 | 130 | 270 |
| 21 | 45.76 | 14.91 | 0.2 | 120 | 140 |
| 22 | 46.74 | 35.73 | 1.1 | 80 | 110 |
| 23 | 47.94 | 12.94 | 1.7 | 130 | 160 |
| 24 | 48.46 | 6.08 | 1.2 | 140 | 160 |
| 25 | 49.36 | 58.92 | 1.7 | 10 | 40 |
| 26 | 49.48 | 69.23 | 0.4 | 140 | 180 |
| 27 | 50.72 | 11.94 | 1.9 | 140 | 170 |
| 28 | 50.97 | 40.65 | 0.1 | 140 | 230 |
| 29 | 52.62 | 35.84 | 1.2 | 130 | 240 |
| 30 | 56.39 | 21.26 | 1.5 | 110 | 210 |
| 31 | 56.57 | 28.74 | 2.0 | 90 | 120 |
| 32 | 57.35 | 39.67 | 1.8 | 80 | 120 |
| 33 | 59.29 | 63.46 | 0.2 | 120 | 270 |
| 34 | 61.22 | 51.65 | 1.0 | 20 | 50 |
| 35 | 61.45 | 55.85 | 1.2 | 50 | 90 |
| 36 | 62.21 | 6.38 | 1.3 | 20 | 110 |
| 37 | 62.42 | 16.49 | 0.7 | 110 | 210 |
| 38 | 63.05 | 43.67 | 0.5 | 120 | 170 |
| 39 | 64.39 | 67.36 | 1.6 | 180 | 240 |
| 40 | 64.64 | 46.42 | 1.7 | 50 | 60 |
| 41 | 65.27 | 25.51 | 0.7 | 130 | 280 |
| 42 | 65.66 | 72.54 | 1.2 | 190 | 290 |
| 43 | 66.43 | 19.36 | 0.2 | 60 | 110 |
| 44 | 71.9 | 19.7 | 1.4 | 140 | 170 |
| 45 | 72.54 | 65.5 | 1.3 | 110 | 220 |
| 46 | 76.75 | 4.19 | 2.0 | 100 | 200 |
| 47 | 77.56 | 49.64 | 0.3 | 80 | 200 |
| 48 | 77.67 | 63.43 | 0.6 | 120 | 280 |
| 49 | 79.11 | 14.71 | 1.8 | 70 | 140 |
| 50 | 79.92 | 42.12 | 2.0 | 110 | 210 |

**Table 4. The parameters in the experiments.**

| **Parameter** | **Description** | **Value** |
| --- | --- | --- |
| *ξ* | Fuel gas mass ratio | 1 |
| *μ* | Fuel oil calorific value (kj/g) | 44 |
| *φ* | Conversion factor coefficient (g/s to l/s) | 737 |
| *ε* | Vehicle’s transmission efficiency coefficient | 0.4 |
| *η* | Engine’s efficiency parameter | 0.9 |
| *k* | Engine’s friction coefficient(kj/r/l) | 0.25 |
| *N* | Engine speed (r/s) | 38.34 |
| *V* | Engine displacement (l) | 4.5 |
| *v* | Vehicle speed (m/s) | 16.67 |
| *ω* | Empty vehicle mass (kg) | 3500 |
| *C_d_* | Vehicle rolling resistance coefficient | 0.01 |
| *C_r_* | Air resistance coefficient | 0.6 |
| *A* | Vehicle’s front area (m^2^) | 7 |
| *ρ* | Air density (kg/m^3^) | 1.2041 |
| *c* | Carbon emission coefficient (kg/L) | 2.778 |
| *C_1_* | GV’s unit transportation cost (Yuan/km) | 1.56 |
| *C_2_* | E V’s unit transportation cost (Yuan/km) | 0.85 |
| *E_max_* | EV’s battery capacity (kwh) | 50 |
| *σ* | EV’s unit power consumption (kwh) | 22 |
| *e* | Electricity emission factor (kg/kwh) | 0.7 |
| *v_i,j_* | Average vehicle speed (km/h) | 60 |
| *s_i_* | Service hours at client *i* (h) | 0.4 |
| *P* | Driver’s hourly salary (Yuan/h) | 20 |
| *Q* | Max vehicle load (t) | 4 |
| *pe* | Unit penalty coefficient for advanced delivery (Yuan/h) | 20 |
| *pl* | Unit penalty coefficient for delayed delivery (Yuan/h) | 40 |
